# Professional identity and moral agency in palliative care: A review

**DOI:** 10.1177/09697330251397446

**Published:** 2025-12-04

**Authors:** Dimitri Létourneau, Luis Enrique Moreno Exebio, Justine Xin Yi Wu

**Affiliations:** 1 Faculty of Nursing, 5622 Université de Montréal, Montreal, QC, Canada; 2 Savoirs partagés Research Centre, Integrated University Health and Social Services Centre of Nord-de-l’Île-de-Montréal (CIUSSS NIM), Montreal, QC, Canada; 3 School of Public Health, 5622 Université de Montréal, Montreal, QC, Canada

**Keywords:** palliative care, nurses, social workers, professional identity, moral agency

## Abstract

**Background: **Nurses and social workers play central roles in palliative care. While moral agency and professional identity have been widely studied, they are rarely examined together, leaving their intersection in palliative care insufficiently understood.

**Aim: **The aim of this integrative review was to identify and analyze the key enablers and barriers influencing the construction of professional identity and the enactment of moral agency among nurses and social workers in the context of palliative care.

**Methods: **The synthesis followed Whittemore and Knafl’s integrative review methodology. 12 databases were queried between February 2022 and November 2023, and a manual search was conducted to identify additional publications. Qualitative analysis was performed using the three concurrent analytic activities proposed by Miles, Huberman, and Saldaña.

**Results:** Of the initial 1448 articles retrieved, 34 were selected through screening, and 4 additional articles were included through manual search, for a total of 38 articles reviewed. For moral agency, the enablers were supportive cultures, relationships with patients and their relatives, and moral capacities and inner dispositions. The barriers identified were restrictive cultures, insufficient resources and workload, and interpersonal value conflicts and moral dissonance. For professional identity, the enablers included making a difference for patients, advocating for patient choices, and patient gratitude as a recursive loop. The main barriers were biomedical dominance and professional devaluation, and value tensions and emotional norms. This review revealed that both concepts are predominantly explored within the nursing literature, are deeply interconnected, and tend to reinforce each other. They also share common enablers and barriers in palliative care settings.

**Conclusion:** The findings suggest the importance of incorporating both concepts into nursing education to support ethical competence in palliative care and to help mitigate the moral distress often experienced by nurses.Hoping this resolves the issue.

## Introduction

Supporting and being present with individuals through the final stretch of life requires embedding humanistic values at the heart of palliative care.^1–3^ Research consistently demonstrates that patients and their relatives strongly desire to be supported by professionals who embrace a humanistic approach manifested in compassionate, person-centered care.^4–6^ Numerous studies have also revealed that the quality of relationships and interactions between healthcare professionals and patients is a cornerstone of patients’ well-being.^7,8^ In palliative care, multiple professional groups are involved, and by the nature of their roles, nurses and social workers are particularly called upon to provide relational and psychosocial support to patients and their relatives.^9,10^

Nurses often experience significant difficulty practicing in alignment with their humanistic ideals due to organizational constraints they struggle to influence, chiefly heavy workloads and the devaluation of humanized care by peers.^11^ Social workers also face similar organizational challenges that hinder their ability to apply their professional ideals in practice.^12–14^ Faced with these obstacles, many professionals find themselves practicing in ways that conflict with the ideals that shaped their professional identity during their education.

In ethics, the capacity of a professional to engage in deliberate and morally meaningful actions, such as practicing in accordance with professional ideals, is referred to as moral agency.^15^ This concept encompasses notions of right and wrong, what is just and unjust,^15^ which can be reflected in a healthcare professional’s conception of what constitutes “good care.” Peter and Liaschenko^16^ emphasize that moral agency is not simply an individual capacity. Instead, they conceptualize it as socially embedded, composed of three interrelated dimensions: identities, relationships, and responsibilities. According to these authors, morality is not a universal or purely rational ideal detached from reality (i.e., not a transcendental notion), but rather a set of practices that are socially and relationally embedded in everyday life. Identities are socially constructed, plural, and relational, shaped by practices and institutions that define specific ways of being.^16^ Together, these three dimensions form a relational and context-sensitive understanding of moral agency, in which the ability to act morally is deeply tied to who one is (identity), what one is responsible for (responsibility), and with whom one is in relationship (relationship).

In education, Bélisle et al. defined professional identity as a dynamic self-representation as a professional (nurse or social worker), based on one’s relationship to oneself, the professional group, and society.^17,18^ The construction of professional identity is one of the three-fold dimensions of professionalization, which is understood as a process of transforming an individual into a professional through the development of competencies and the internalization of professional culture.^17,19^ Professionalization is seen as a process that begins before formal education, takes shape during academic training, and continues into professional practice (post-professionalization).^17,18^ It is also posited that this professionalization trajectory is embedded within curricular, pedagogical, institutional, social, and political contexts, a view that aligns well with the relationship between moral agency and the social context emphasized by Peter and Liaschenko.^16^

For various reasons, the enactment of moral agency among nurses and social workers may be constrained, leading to disruptions in their professional identity.^20^ For instance, during the COVID-19 pandemic, practices such as the rationing of protective equipment, resource allocation under conditions of scarcity, and visitor restrictions^21–23^ created moral tensions, as professionals often felt they were acting in ways they perceived as not being in their patients’ best interest.^24^ Collectively, these changes have deeply challenged the notion of “good” palliative care, generating moral paradoxes and contradictions for professionals. When professionals are unable to reconcile their ideals with their practice and cannot act as moral agents, they are at risk of experiencing cognitive dissonance, disillusionment, and moral distress,^25–28^ a phenomenon that shares many consequences with professional burnout.^12,29^ These harmful effects can lead to job dissatisfaction and professional attrition,^30,31^ and can also impact patients and families.^32^ In palliative care contexts, this may diminish the quality of interactions between healthcare professionals and patients and families, such interactions being particularly crucial.^4–6,33,34^ These findings highlight the importance of focusing on professional identity and moral agency among nurses and social workers in such contexts. These studies also underscore that while these concepts have been moderately examined within the disciplines of nursing and social work, particularly regarding the consequences of their erosion, little research has synthesized the factors that facilitate or hinder their development. This knowledge gap is particularly significant in the context of growing demands in healthcare environments where resources are often limited, and ethical tensions are pervasive. A knowledge synthesis offers the opportunity to bring together the most salient enablers and barriers of each concept, providing a clearer understanding of the levers that can be activated to support their development.

## Aim

This integrative review aimed to identify and analyze the key enablers and barriers influencing the construction of professional identity and the enactment of moral agency among nurses and social workers in the context of palliative care.

## Method

An integrative review based on Whittemore and Knafl^35^ methodology was used. It was chosen to inform this review because it allows for the synthesis of various papers, including both empirical qualitative and quantitative research and theoretical/discussion articles. Our preliminary search on databases had revealed a diversity of paper types, both for moral agency and professional identity, hence why we chose an integrative review method. Whittemore And Knafl^35^ methodology involves five recursive stages comprising (1) problem identification, (2) literature search, (3) data evaluation, (4) data analysis, and (5) presentation.

### Database literature search strategy

A disciplinary librarian was consulted to develop the search strategy. The 12 databases that were queried on February 2022 by one team member (JXYW) are as follows: CINAHL, MEDLINE, Embase, Social Work Abstracts, Social Sciences Abstracts, Social Services Abstracts, Sociological Abstracts, Dissertations and Theses Global, Web of Science, JBI EBP Database, and EBM Reviews - Cochrane Database of Systematic Reviews. When these databases were first queried, we had included publications published after 2012 (a 10-year range), which yielded 2288 records after duplicate removal. As only two researchers (DL and JXYW) were working on the project at the time, we anticipated that this number would be too extensive for the available resources. Therefore, for pragmatic reasons, we decided to restrict the search to publications from 2017 onward (a 5-year range).

The search strategy included four main concepts comprising keywords and subject headings, which are as follows: (1) moral agency, (2) professional identity, (3) palliative care, and (4) nursing and social work. To keep our literature up-to-date, we repeated our initial search strategy with the same 12 different databases in November of 2023. To further ensure the currency of our review before publication, one team member (DL) conducted a forward citation search in October 2025 for each paper that met the inclusion criteria. However, no additional relevant publications were identified. For a detailed account of our search strategy, including keywords and search equations, please refer to Supplemental Material 1.

### Inclusion and exclusion criteria and screening process

To be eligible, the following inclusion criteria were used: (1) written in English or French; (2) published after 2017; (3) the population must consist exclusively of nurses or social workers, or at least 50% of them; (4) the content must clearly relate to enablers/barriers to moral agency or professional identity; and (5) the palliative care component must constitute at least 50% of the content and be explicit. For this last criterion, we did not limit our search to the palliative care unit, because palliative care is an approach aiming at improving the quality of life of individuals facing life-threatening illnesses.^36^ This approach can be integrated within the community and across various hospital wards. Moreover, we acknowledge that the relationship between palliative care and medical assistance in dying (MAiD) is debated in the literature. Perspectives vary: some draw parallels between the person’s experience of MAiD and other end-of-life experiences,^37^ other consider them distinct entities,^38^ while some place them on a continuum of end-of-life care.^39^ For this review, we chose to include publications discussing MAiD, as we anticipated potential intricacies related to moral agency and professional identity within the context of palliative care.

Publications were excluded based on the following criteria: (1) research protocols, commentaries, editorials, tool or measurement scale development, or conference abstracts; (2) full-text unavailable; (3) papers with unclear population definitions; (4) papers focusing on students rather than graduated professionals; (5) papers not clearly addressing moral agency or professional identity; (6) papers with ambiguous palliative care contexts; (7) older papers serving as the basis for subsequent included publications; and (8) more recent papers presenting already published results from previous included publications. Moreover, literature reviews were not excluded, as we considered that their synthesized findings could enrich our analysis by providing complementary insights beyond those of the individual primary studies they summarized. To avoid potential data overlap, specific measures were implemented, as described in the data extraction section.

Publications from both batches (February 2022 and November 2023) underwent a three-phase screening process using Microsoft Excel and Covidence: (1) title and abstract review, (2) keyword search and preliminary scan of the full text, and (3) full-text review. A calibration exercise was first conducted among team members using a small sample of papers (*n* = 15) to ensure consistency. The first two screening phases were carried out independently by two reviewers, with discrepancies resolved through discussion. The third phase, involving full-text assessment, was conducted by a single reviewer, with any remaining disagreements adjudicated by the primary investigator (DL).

Forward and backward cascade referencing methods (manual searching) were also performed on the included articles to identify references that could have been missed through queries on the databases. The same inclusion and exclusion criteria were applied to all publications identified through these methods.

### Data extraction and quality assessment

The processes of data extraction and quality appraisal followed the same logic as the search strategy. A calibration exercise was first conducted on a sample of 10 eligible publications, deliberately selected to reflect different types of articles. Subsequently, data extraction and quality assessment were performed by a single reviewer for each publication.

Data was extracted in a Microsoft Excel spreadsheet using categories (i.e., key results related to moral agency and professional identity). Extracted material included integral excerpts from the publications such as qualitative or quantitative findings from empirical studies, synthesized results from literature reviews, and relevant statements from theoretical articles that addressed our research questions. This spreadsheet was synthesized into a table presented in Supplemental Material 2.

After completing the data extraction process, we examined the list of studies synthesized in each literature review and compared them with the publications retained in our corpus to identify any potential overlap. This verification revealed that three literature reviews cited five of our primary studies; however, only two of these were actually used as primary data sources within a single literature review. Further examination confirmed that none of our excerpts from that review were directly related to these two primary studies, thereby confirming the absence of duplication across data sources.

Three quality appraisal tools were used, depending on publication type: (1) the Mixed-Methods Appraisal Tool (MMAT) developed by Hong^40^ for empirical studies, (2) the Joanna Briggs Institute (JBI) Checklist for Systematic Reviews and Research Syntheses^41^ for literature reviews, and (3) the JBI Checklist for Text and Opinion^42^ for theoretical papers. Because the appraisal tools used in this review rely on categorical responses (e.g., “yes,” “no,” and “unclear”) rather than numerical scoring, the overall quality of each publication was determined through a global judgment based on the number and consistency of positive appraisals. Each publication was then classified as having “low,” “average,” or “high” methodological quality. Importantly, this quality assessment was intended to inform the interpretation of findings, not to exclude publications from the review.

### Data analysis and synthesis

Our analysis was guided by the three concurrent analytic activities proposed by Miles, Huberman, and Saldaña^43^: data condensation, data display, and conclusion drawing and verification. We used the excerpts previously identified during data extraction as the foundation for interpretation. We returned to the original articles to review these excerpts in context, to ensure that the surrounding paragraph or sentence was considered in the interpretation. An additional column was added to the Microsoft Excel spreadsheet to document emerging interpretations, complemented by entries in a reflective journal. To integrate data from different types of publications, we ensured that the common analytical ground consisted of textual forms. When excerpts were quantitative in nature, we used the additional column to interpret what the findings represented in terms of enablers or barriers related to moral agency or professional identity, thereby transforming them into a textual form.^35^ Each excerpt was assigned a tentative code, based on our theoretical framework related to moral agency^16^ and professional identity.^17^ Through iterative team discussions among team members (DL, JXYW, and LEME), we examined thematic connections and developed categories based on the coded excerpts. This process allowed us to organize our emerging interpretations according to the enablers and barriers of moral agency and professional identity. We also paid close attention to potential relationships and intersections between these two concepts.

## Results

**Figure 1.**
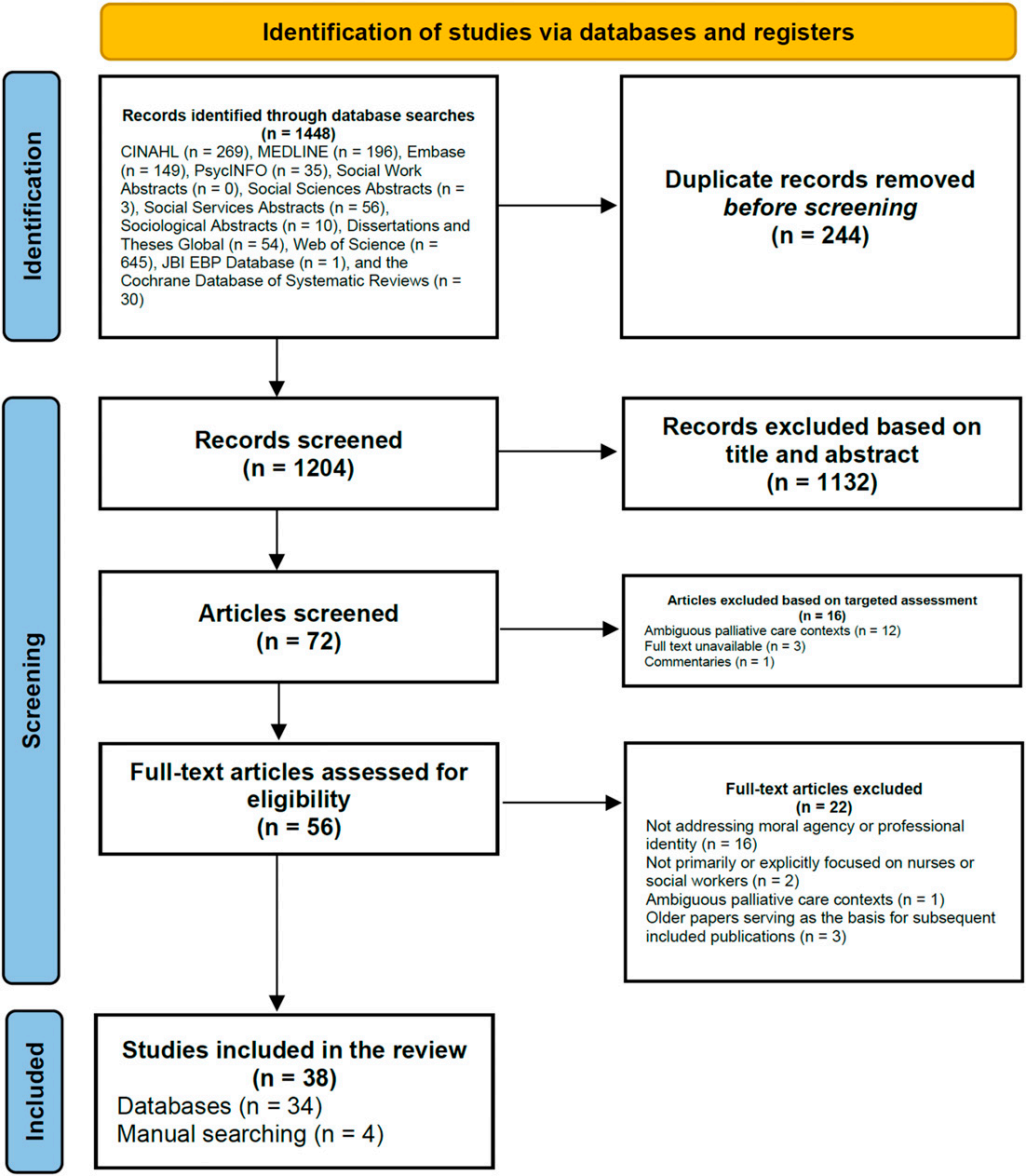
Flow diagram of article selection, based on searches conducted in February 2022 and November 2023 (modified PRISMA flow diagram^44^).

Figure 1 presents the integrated PRISMA flow diagram combining the two rounds of database searches conducted in February 2022 and November 2023. A total of 1448 records were identified across 12 databases. One database (Social Work Abstracts) yielded no relevant records. After removing 244 duplicates, 1204 records remained. Of these, 1132 were excluded based on title and abstract screening. The remaining 72 reports were sought for retrieval, with 16 subsequently removed following browsing and targeted assessment. A total of 56 full-text articles were assessed for eligibility, leading to the inclusion of 34 articles. An additional 4 relevant articles were identified through manual searches, resulting in a final sample of 38 articles included in the review.

### Characteristics of included articles

The 38 included articles originated from 17 countries across five continents, based on the first author’s country of affiliation (see Table 1). North America was strongly represented, particularly Canada (*n* = 15) and the United States (*n* = 5). Europe contributed 11 articles, while Asia, Oceania, and Africa were more modestly represented. Of the included articles, 27 were empirical studies, 7 were literature reviews, and 4 were theoretical reflections (see Table 2). Various qualitative methodologies predominated, accounting for 25 of the 27 empirical studies.

**Table 1.** Geographic distribution of articles by country and continent.

America (*n* = 20)	Europe (*n* = 11)	Asia (*n* = 3)	Oceania (*n* = 3)	Africa (*n* = 1)
Canada (*n* = 15)	Belgium (*n* = 1)	Korea (*n* = 1)	Australia (*n* = 2)	Ethiopia (*n* = 1)
Beuthin et al.^45^	Bellens et al.^65^	Lim and Kim^76^	Panozzo et al.^79^	Gebreheat and Teame^82^
Carnevale^46^	Italy (*n* = 1)	Taiwan (*n* = 1)	Reed et al.^80^	
Dormand and Bouchal^47^	Albanesi et al.^66^	Prompahakul and Epstein^77^	New Zealand (*n* = 1)	
Elmore et al.^48^	France (*n* = 1)	Thailand (*n* = 1)	Mowat et al.^81^	
Fantus et al.^49^	Laugerat et al.^67^	Ko et al.^78^		
Fortier^50^	Greece (*n* = 1)			
Funk et al.^51^	Voultsos et al.^68^			
Kopchek^52^	The Netherlands (*n* = 2)			
Ma et al.^53^	Lokker et al.^69^ van den Bosch et al.^70^			
McMillan et al.^54^	Norway (*n* = 2)			
Peter et al.^55^	Ramvi and Ueland^71^			
Rodney^56^	Jerpseth et al.^72^			
Variath et al.^57^	Spain (*n* = 1)			
Wright et al.^58^	Falcó-Pegueroles et al.^73^			
Wright et al.^59^	Sweden (*n* = 1)			
United States (*n* = 5)	Glasdam et al.^74^			
Dressler et al.^60^	United Kingdom (*n* = 1)			
Hold^61^	Bradshaw et al.^75^			
Meeker and White^62^				
O'Mathúna et al.^63^				
Rainer et al.^64^

**Table 2.** Classification of included articles by type and methodological approach.

Categories	Subcategories
Empirical studies (*n* = 27)	Quantitative (*n* = 1)
	Qualitative (*n* = 25)
	Mixed-methods study (*n* = 1)
Literature reviews (*n* = 7)	Concept analysis (*n* = 1)
	Integrative review (*n* = 3)
	Qualitative meta-synthesis (*n* = 1)
	Scoping review (*n* = 1)
	Systematic review (*n* = 1)
Theoretical articles (*n* = 4)	Practice narrative (*n* = 1)
	Theoretical exploration (*n* = 3)

In terms of populations, 30 articles (79%) focused exclusively on nurses, only 1 (3%) focused solely on social workers,^49^ and 7 (18%) included mixed samples, primarily composed of nurses, social workers, physicians, psychologists, and administrators.

Of the 38 articles, 15 explicitly addressed the concept of moral agency, especially those from Canada and the United States, while 3 included the concept of professional identity, and only 1 explicitly referred to both. The remaining 19 articles did not explicitly mention these concepts, but their content reflected themes related to one or both, as interpreted in light of the theoretical underpinnings of our study.^16,17^

In terms of quality appraisal, most publications (*n* = 31) were appraised as having high quality, while 6 were rated as average and 1 as low. The reasons why certain publications were appraised as average included, for example, the absence of justification for limiting the search strategy to nursing science databases despite a broader review aim^47^ or a lack of clarity regarding the role of quantitative data in a mixed-methods study.^80^ The only paper^69^ appraised as low did not justify its use of a secondary analysis of interviews conducted a decade earlier for a different research objective, raising concerns about the adequacy of the data. Additionally, there was no indication that thematic saturation had been reached, and the age of the data may have limited the applicability of the findings to current practice. Indeed, this study explored nurses’ experiences of morally distressing situations related to the practice of palliative sedation in the Netherlands,^69^ where the Royal Dutch Medical Association (KNMG) has issued new guidelines^83,84^ on such practices in recent years (2021–2022). These updated guidelines place greater emphasis on interprofessional collaboration and proactive communication with patients and families, which may influence how nurses experience moral distress in current practice.

### Enablers to moral agency

#### Supportive culture

A supportive culture facilitates moral agency by creating the conditions necessary for healthcare professionals to recognize, deliberate on, and act according to their ethical commitments. This culture rests on three key components: interprofessional support, the formation of a moral community, and a climate of ethical dialogue rooted in patient-centered care.^53^ Interprofessional support fosters moral agency by enabling collaborative decision-making that aligns with shared values and individualized care goals. Through mutual respect and shared responsibility, clinicians can navigate complex ethical situations with more confidence, promoting actions grounded in the dignity of patients.^53,56^ Beyond support among professionals, this culture also encompasses the provision of emotional guidance and counseling to patients’ families, such actions reflecting and reinforcing the clinician’s role as a moral agent engaged in relational and context-sensitive care.^55^

The establishment of a moral community^50^ and a culture of ethical discourse fosters the development of moral identity and a shared understanding of ethical responsibilities among healthcare professionals.^15,53,58^ These elements create the conditions necessary for clinicians to recognize ethical tensions, reflect collectively, and act in ways that are consistent with their professional integrity, even when such actions challenge institutional or cultural norms. In palliative care settings, where ethical tensions are frequent and decisions are deeply value-laden, nurses are uniquely positioned to enact and support moral agency. Drawing on their close relational proximity to patients and families, they serve as key facilitators of consensus-building, mediators of value conflicts, and advocates for care that upholds the dignity, preferences, and lived experiences of those at the end of life.^50,62^ By remaining present in morally complex situations and creating spaces for dialogue and shared decision-making, nurses demonstrate a form of relational moral agency grounded in both clinical insight and ethical commitment.

#### Relationships with patients and their relatives

Establishing meaningful relationships with patients and families is central to the exercise of moral agency in palliative care. Good communication and a deep understanding of the patient foster proximity, trust, and effective teamwork.^61^ These relationships enable nurses to engage with the moral complexity of each situation, as they continuously negotiate the boundaries between their personal and professional selves.^66^ This negotiation is a key element of moral agency, requiring reflection and integrity to maintain ethical responsiveness without overstepping relational boundaries.

The nurse–patient relationship, grounded in honesty, respect for personhood, and a commitment to patient autonomy, provides a moral space where care is co-constructed in alignment with patients’ values and lived experiences.^62^ This relational foundation supports the nurse’s capacity to recognize what is ethically at stake and to act accordingly, especially in the face of conflicting expectations.

Acquiring moral knowledge from the family further enhances nurses’ moral agency, by helping them understand what constitutes a “good death” in each unique context. Since interpretations of a good death vary widely and can evolve throughout the dying process,^48,59,61^ nurses must remain attuned to the shifting moral landscape shaped by the patient’s and family’s values. This relational attunement empowers nurses to mediate ethically charged decisions, adapt care plans sensitively, and act with moral clarity in service of a dignified end-of-life experience.

#### Moral capacities and inner dispositions

The exercise of moral agency in palliative care requires not only contextual awareness and ethical knowledge but also a set of internal dispositions that support sustained moral engagement. Among the most critical qualities, skills, and attitudes are the ability to engage in ethical discussions, the capacity for moral introspection and discernment, the willingness to resist institutional norms when they conflict with ethical commitments, and the sense of professional fulfillment that arises from having “done the right thing.”^51,81^ These dispositions include emotional intelligence and moral sensitivity (particularly the awareness of how one’s actions affect the grief of families) as well as a belief in one’s own potential to make morally sound decisions.^76,80^ This moral self-confidence is especially important in ethically ambiguous situations where external guidance may be limited or contested. Together, these attributes reflect a form of embodied and relational moral agency, enabling clinicians to act from a place of ethical conviction, grounded in emotional and reflective capacities that sustain moral presence in the face of suffering and systemic constraints.^50^

Further capacities such as moral clarity and moral reflectiveness deepen nurses’ ability to identify, deliberate on, and navigate ethical challenges in palliative contexts.^58^ Moral clarity, often supported by ethics education,^69,76^ enables nurses to articulate their values and align them with their professional role. Moral reflectiveness supports the ongoing negotiation of ethical uncertainties^59^ (i.e., morally complex questions without clear-cut answers). Together, these capacities strengthen the clinician’s ability to act with integrity in the face of ambiguity, constraint, or conflict, and thereby uphold a morally responsive practice.

### Barriers to moral agency

#### Restrictive cultures

The exercise of moral agency in palliative care is often constrained by systemic and organizational barriers that limit nurses’ capacity to act in accordance with their ethical commitments. One of the most frequently reported obstacles, both in Canada and internationally, is the rigidity of institutional protocols and regulations governing patient care.^47,57^ These often reduce nurses’ discretionary space to respond ethically in complex and evolving situations (particularly during health crises such as the COVID-19 pandemic) where standardized procedures may conflict with the moral demands of individualized care.^54,70^ These constraints compromise nurses’ moral space and undermine their ability to exercise situated judgment, a core component of moral agency.^75^

Hierarchical structures within healthcare institutions present another major barrier.^77^ When authority is concentrated in rigid professional silos, nurses, despite their direct and sustained contact with patients, may find their ethical perspectives dismissed or overridden by those in higher positions.^68,69^ This epistemic subordination limits the moral agency of nurses by denying them recognition as moral agents capable of ethical reasoning and decision-making. It creates a disjunction between ethical perception and ethical action.

Additional barriers include the lack of recognition for nurses’ expertise in palliative care,^71^ particularly by external stakeholders such as administrators or physicians.^61^ Inadequate or dysfunctional teamwork,^65^ and the absence of professional solidarity further erode the collective foundation required for shared moral deliberation and support. Without collaborative moral spaces, moral agency becomes more difficult to sustain.^15^

Cultural dynamics such as intimidation and fear of retaliation also directly threaten nurses’ moral agency. In environments where nurses fear losing their jobs for voicing ethical concerns^50^ or invoking conscientious objection,^68^ moral courage is stifled, and ethical silence becomes a survival strategy.

In the COVID-19 pandemic context, inconsistencies between sanitary protocols and the values of palliative care^67,73^ placed nurses and other providers in morally distressing situations. Infection prevention and control (IPC) measures often prevented clinicians from acting in accordance with their ethical commitments, for instance, by restricting family visits or delaying communication about a patient’s imminent death.^75^ These practices directly conflicted with healthcare workers’ deeply held values of transparency, presence, and relational care. Furthermore, clinicians were sometimes unable to honor patient wishes, for instance, the desire to be discharged home, due to public health restrictions.^75^ The inability to fulfill these ethically significant requests undermined their sense of moral responsibility and capacity to support patient autonomy. Even the physical barriers introduced by personal protective equipment (PPE) made it more difficult to express empathy and build trusting relationships, which are foundational to palliative care.^75^ These examples illustrate how the COVID-19 pandemic not only introduced logistical challenges but also eroded the ethical space and the relational conditions necessary for moral agency, often leaving clinicians ethically constrained and emotionally isolated in their practice.

These restrictive cultural dynamics, taken together, compromise nurses’ ability to perceive moral salience, deliberate ethically, and act with integrity, the very core of moral agency as relational, reflective, and embedded in the everyday realities of care. In contrast with supportive environments that nurture moral agency, restrictive cultures create moral climates where ethical action is constrained, silenced, or delegitimized.

#### Insufficient resources and workload

Chronic work overload and burnout are recurring themes among both social workers^49^ and nurses^56,82^ and are often considered intrinsic to palliative care practice. These conditions affect not only the physical and psychological well-being of healthcare providers but also their capacity to remain ethically attuned and morally present in their daily interactions with patients and families. When nurses are overwhelmed, their ability to reflect, deliberate, and engage in relational ethical care is significantly diminished.

A related structural barrier is the persistent shortage of healthcare personnel,^49,51,56,64^ which leads to increased workloads and compromises the possibility of delivering compassionate care. The lack of sufficient time^47,50,51,65^ further compresses the moral space available for nurses to consider the values, beliefs, and unique needs of patients. Instead, ethical decision-making becomes rushed or fragmented, undermining the relational and reflective dimensions of moral agency.

These systemic pressures are further exacerbated by the unavailability of adequate resources,^49,57^ often due to budgetary restrictions. In some cases, even basic protective equipment or essential medical supplies are lacking,^82^ which can place nurses in morally distressing situations where they may have to choose between personal safety and patient care.

Collectively, these structural constraints do more than impair operational efficiency: they erode the moral infrastructure of care. They reduce the freedom, time, and relational space necessary for nurses to perceive morally salient moments, reflect ethically, and act in ways aligned with their professional values.

#### Interpersonal value conflicts and moral dissonance

Moral agency is rooted in the ability of healthcare professionals to act in accordance with their ethical commitments and to navigate value-laden situations with integrity. However, in palliative care, nurses often face significant tensions when their own values come into conflict with those of patients, families, or institutional mandates. These value conflicts can take many forms and often result in moral dissonance, where nurses recognize what feels ethically appropriate, but feel constrained or unable to act on it. One frequent example is the inability to challenge medical orders that conflict with the nurse’s sense of what is ethically “right” or compassionate.^63^ These situations can create moral distress and undermine the nurse’s agency by reinforcing a model of care where obedience overrides moral judgment.

Other forms of value discrepancy emerge from differences between nurses’ personal or cultural beliefs and the values of patients or families.^56,61,81^ These tensions may be particularly acute around end-of-life issues such as MAiD, where religious or cultural values may diverge sharply from patients’ expressed wishes.^57,59^ Nurses may also feel personally or culturally unprepared to discuss death openly, which can act as a barrier to meaningful communication and relationship-building.

When clinicians cannot reconcile these value differences, their moral clarity and sense of coherence can be disrupted, weakening their capacity to respond authentically and ethically to patient needs. This can ultimately diminish the relational and reflective components of moral agency, and leave nurses feeling morally isolated or emotionally fragmented in their practice.

### Enablers to professional identity

#### Making a difference for patients

The aspiration to make a meaningful difference in the lives of patients constitutes a central foundation for the development of nurses’ professional identity.^55^ This desire, often expressed through daily acts of care, allows nurses to perceive their role as both impactful and ethically significant. Their sense of self is shaped not only by what they do, but by the meaning they attach to their actions in the context of patient care.

Through sustained relationships with patients and families, nurses develop a deeper understanding of the experiences and needs of those they care for.^61^ These interactions reinforce a feeling of professional purpose and enable nurses to position themselves as distinct within the healthcare team, not only in function, but in the quality of their engagement and presence with patients.^58^ This sense of distinctiveness reinforces professional identity by allowing nurses to see their personal values such as compassion, respect, or the desire to help, reflected in the care they provide and the positive outcomes they help generate.

The ideal of the “good nurse” is a socially constructed and enduring figure within the nursing profession, one that remains closely linked to the capacity to make a meaningful difference in patients’ lives.^55^ This ideal acts as a normative reference point, shaping how nurses see themselves and how they strive to act, even within contemporary healthcare systems that are often governed by efficiency, technology, and standardization. Professional identity is thus not solely derived from formal roles or job descriptions, but also from the ethical and emotional weight that nurses assign to their daily practices. The way they engage with patients, respond to suffering, and uphold values such as dignity and compassion becomes central to how they define themselves as professionals.

#### Advocating for patient choices

The involvement of nurses in MAiD underscores a key dimension of their professional identity: the commitment to respecting patient autonomy and accompanying individuals with dignity at the end of life.^57,59^ While MAiD is sometimes framed differently from “traditional” palliative care approaches, it nonetheless offers nurses an opportunity to affirm their role as advocates for patient choices and to remain aligned with their professional values of compassion, presence, and nonjudgmental support.

In this context, nurses engage in ongoing reflective processes to examine the ethical and emotional dimensions of participating in MAiD. These reflections are not peripheral because they are central to how nurses navigate the complexities of their role and make sense of their professional self. Upholding a patient’s wish to receive MAiD, when done within a framework of respect for autonomy, reinforces nurses’ sense of moral coherence and strengthens their identity as professionals who support individuals’ right to make deeply personal decisions at the end of life.

Moreover, the ability to support a patient-defined understanding of a “good death” allows nurses to position themselves as ethical and relational professionals, capable of acting not only in accordance with institutional or legal frameworks but also in ways that uphold the personal values and end-of-life preferences expressed by patients. This advocacy role contributes directly to the construction of professional identity by reaffirming the nurse’s place at the intersection of ethics, care, and individualized decision-making.^48^

#### Patient gratitude as a recursive loop

Professional identity is also shaped by how patients respond to care, especially when they express gratitude in recognition of a meaningful difference made in their experience. When patients explicitly acknowledge the relief brought by nursing care, this feedback acts as a form of confirmation that reinforces the nurse’s sense of ethical purpose and professional legitimacy.^55^

These moments create a recursive loop: the nurse’s actions lead to an impact that is recognized and acknowledged by the patient, which in turn reinforces the nurse’s identification with their professional role. This recognition helps ground identity not only in tasks or competencies, but in the relational significance of care. In this way, gratitude serves as a form of reflective feedback that affirms the value of nursing practice and contributes to the ongoing construction of a professional identity.^55^

### Barriers to professional identity

#### Biomedical dominance and professional devaluation

The predominance of the biomedical paradigm in many healthcare environments creates significant tensions for nurses whose practice is grounded in more humanistic and relational ideals, particularly within palliative care. When institutional priorities or the values of other professionals are guided primarily by curative goals and technical efficiency, nurses may find themselves unable to fully exercise their roles, especially in situations where ethical presence, listening, or comfort are central to patient care.^61^ This disconnect between institutional logics and professional values can undermine nurses’ sense of coherence and legitimacy, contributing to identity fragmentation.

In parallel, few studies report that nurses often experience a devaluation of their professional contributions, particularly when their competence is minimized by physicians or by organizational cultures that fail to acknowledge the complexity of nursing judgment.^71^ When nursing expertise is framed in reductive or instrumental terms (limited to task execution or emotional labor), it becomes difficult for nurses to maintain a strong and integrated sense of their professional identity. The feeling of being perceived as peripheral or replaceable weakens professional confidence and blurs the boundaries of the role.

Together, these factors (dominant biomedical framing and lack of professional recognition) create a working environment that challenges the legitimacy and self-definition of nurses. They obscure the ethical, relational, and clinical contributions that are foundational to nursing identity, especially in palliative care contexts where the meaning of care extends well beyond technical intervention.

#### Value tensions and emotional norms

Professional identity can be destabilized when nurses face ethical tensions within clinical decisions, such as MAiD or whether to initiate palliative sedation. Some nurses have described feeling that sedation was urgently needed to relieve suffering, while physicians judged it too soon; others felt sedation was administered without exhausting other options.^59^ These situations create dissonance between ethical conviction and clinical action, challenging nurses’ sense of coherence. This is further complicated by professional norms that encourage nurses to maintain a certain emotional distance, that is to stay “in control,” avoid showing grief, and limit personal expression.^51^ While often framed as professional behavior, this emotional restraint can act as a barrier to the empathic connection that is particularly central in palliative care. The tension between staying detached and engaging authentically can undermine nurses’ ability to build a professional identity rooted in humanistic and relational values.^66^

Yet, when nurses are supported in engaging in critical reflection on these tensions, they are better able to integrate them into a resilient and ethically grounded professional identity. In this way, when critical reflection is supported, it can transform ethically challenging situations into opportunities for growth. Conversely, the absence of such reflection, due to lack of time, space, or support, can act as a barrier to identity construction, leaving nurses with unresolved tensions and a fragmented sense of professional self.^59^

## Discussion

This integrative review highlights several key findings regarding the articulation of moral agency and professional identity in the context of palliative care.

The first finding concerns the disciplinary imbalance in the literature. Most studies originate from the discipline of nursing, with very limited contributions from social work. The sole article addressing the relationship between moral distress and moral agency in social workers^49^ emphasizes the need to integrate ethical reflection and moral distress into professional training to mitigate frustration and preserve professional integrity. This was particularly salient in palliative care, where moral tensions are frequent and complex.

Secondly, the review reveals that moral agency and professional identity are often studied separately, or in association with other constructs (i.e., moral distress, resilience, or burnout), but rarely in direct relation to palliative care. This siloed approach fails to recognize that these two dimensions are interconnected and, in practice, often mutually reinforcing. Moral agency enables nurses to act ethically within the complexity of palliative care, while professional identity provides the ethical grounding and sense of self needed to sustain such action. For instance, Fortier^50^ found that organizational constraints such as excessive workloads, insufficient staffing, and lack of time, undermine nurses’ ability to exercise moral agency, thereby contributing to moral distress and weakening their sense of professional fulfillment. Meeker and White^62^ demonstrate that nurses’ moral agency, expressed through their support of families during transitions to comfort care, is rooted in their relational proximity and ethical responsiveness. These morally engaged actions do not only benefit patients and families; they also reinforce the nurse’s sense of identity as a professional who upholds humanistic values, ensuring dignity and being present with patients throughout their end-of-life journey. In this way, acting as a moral agent becomes a means through which nurses consolidate who they are as professionals.

In parallel, the reviewed literature suggests that professional identity is constructed through everyday engagement with ethically meaningful care, particularly in how nurses interpret and navigate their roles in relation to others. Peter et al.^55^ highlight that the gratitude expressed by patients contributes to nurses’ moral identity and sense of professional purpose. This form of recognition reinforces nurses’ sense of having fulfilled their professional purpose, thereby strengthening their professional identity. In palliative care, where the outcomes of care are at times intangible and deeply interpersonal, this dynamic becomes especially salient: identity is not shaped solely by technical proficiency, but by the emotional resonance and ethical significance of the care provided. Conversely, Beuthin^45^ illustrates that practices such as MAiD can blur the boundaries between personal and professional identity. These ethically charged contexts often prompt deep reflection and, in some cases, give rise to moral distress when nurses struggle to reconcile institutional demands, personal beliefs, and their understanding of professional responsibility.^59^

A third key finding is the overlap between enablers and barriers to moral agency and professional identity. This convergence suggests that similar structural, relational, and individual factors can either support or hinder both dimensions. For example, supportive interpersonal relationships, interprofessional collaboration, and ethical dialogue foster the exercise of moral agency^50,64^ and simultaneously sustain professional identity through recognition, validation, and relational presence.^55,57,58^ Similarly, internal capacities such as moral sensitivity, emotional intelligence, and moral clarity support both moral action and the integration of professional values into a coherent self-concept.^16,51^ Critical reflection, when actively supported, allows nurses to navigate tensions between personal beliefs and professional expectations (particularly in ethically complex practices such as MAiD) while also reinforcing their identity as ethical and relational caregivers.^48,59^

Conversely, many of the barriers that hinder moral agency also obstruct the construction of professional identity. Restrictive institutional cultures,^47,68^ hierarchical authority,^71^ and lack of recognition^50,53^ erode nurses’ autonomy and obscure their moral contributions, undermining their ethical decision-making capacity and identity affirmation. Structural constraints such as staff shortages,^51^ time pressures,^77^ and insufficient resources^51^ limit nurses’ ability to provide dignified, individualized care, thus weakening both their moral integrity and sense of professional fulfillment.^56^ Furthermore, value conflicts between nurses’ ethical convictions and institutional or patient/relative expectations often give rise to moral distress, which, in turn, erodes professional identity and motivation.^69,75^ These intertwined challenges highlight the need for organizational and educational strategies that simultaneously support both moral agency and professional identity to ensure ethical, sustainable nursing practice in palliative care.

These findings support the view that moral agency and professional identity co-emerge through practice, and that both are sustained (or threatened) by similar structural, relational, and contextual conditions. A common thread linking the two is the relational nature of nursing care, which consistently appears as a core element in how nurses act ethically and understand themselves professionally. The nurse–patient relationship, grounded in humanistic ideals, offers the space where values are enacted and identities are affirmed.

In line with Bélisle’s^17^ perspective, identity is not a fixed state, but a continuous process of self-definition shaped through relationships, values, and practice contexts. Likewise, following Peter and Liaschenko,^16^ moral agency is not merely the capacity to choose ethically, but the ability to act with integrity within relationally and institutionally constrained spaces. The proximity nurses maintain with patients and families, especially in palliative care, grounds both their ethical responsiveness and their evolving sense of professional self.

### Limitations and strengths

One of the limitations of this review lies in the inherent challenge of clearly distinguishing between barriers and facilitators related to the two core concepts. Half of the included papers (*n* = 19) did not focus specifically on moral agency or professional identity, which complicated the interpretation process. Moreover, because the inability to enact moral agency is closely linked to the experience of moral distress, the research team noted that the boundaries between enablers and barriers of moral agency and factors contributing to moral distress were sometimes blurred, despite team discussions. It is therefore possible that some of the enablers and barriers identified as relevant to moral agency may also reflect conditions that influence moral distress.

In terms of strengths, the originality of this review lies in its integration of concepts from two distinct disciplines (ethics and education). To our knowledge, this dual-disciplinary lens has not been applied in previous reviews on this topic. While we anticipated identifying multiple links between moral agency and professional identity, the limited number of publications addressing professional identity directly made this objective more challenging to achieve.

## Conclusion

This integrative review identified key enablers and barriers to the moral agency and professional identity of nurses and social workers in palliative care. These two concepts are deeply interconnected and mutually reinforcing; thus, the factors that support or hinder one often affect the other. Notably, the findings reveal that barriers, whether structural, relational, or institutional, are particularly prevalent. To prevent these professionals from experiencing moral distress when their values are challenged, it is essential to act upstream. Educational programs could aim to strengthen their ethical capacities while consolidating their professional identity during training, enabling them to better anticipate and navigate these barriers. Such preparation can support them in enacting their moral agency in ways that align with their humanistic professional ideals and foster a coherent, resilient sense of identity. Future studies could focus on strategies to reinforce these enabling factors and address the obstacles that impede ethical and identity-related development in palliative care settings.

## Supplemental Material

Supplemental Material - Professional identity and moral agency in palliative care: A review Supplemental Material for Professional identity and moral agency in palliative care: A review by Dimitri Létourneau, Luis Enrique Moreno Exebio, and Justine Xin Yi Wu in Nursing Ethics.
