# Supplementary material for: Professional identity and moral agency in palliative care: A review
Source: Nurs Ethics. 2025 Dec 4;33(3):691–709. doi: 10.1177/09697330251397446 (PMC13103347; doi:10.1177/09697330251397446)
Supplement: Supplemental Material - Professional identity and moral agency in palliative care: A review [file sj-pdf-1-nej-10.1177_09697330251397446.pdf]

**Supplementary Material 1: Search Strategy**

| Concepts | Professional Identity | OR | Moral Agency | Palliative Care | Nurses and Social Workers |
|---|---|---|---|---|---|
| Keywords | -Professional identity<br>-Professionalization<br>-Professionalism education<br>-Professional formation<br>-Identity formation<br>-Identity construction<br>-Professional education<br>-Professional training<br>-Professional self-concept<br>-Professional values<br>-Professional image<br>-Professional comportment<br>-Professional competence<br>-Professional conduct<br>-Professional role<br>-Nursing role<br>-Social worker role<br>-Professional ethics<br>-Nursing ethics<br>-Social work ethics<br>-Professional dignity<br>-Nurses identity<br>-Social workers identity<br>-Ethical identity<br>-Ethical comportment<br>-Ethical practice<br>-Ethical values | | -Moral agency<br>-Moral agent<br>-Moral identity<br>-Moral philosophy<br>-Moral psychology<br>-Moral conflict management<br>-Moral conflict resolution<br>-Moral judgement<br>-Moral values<br>-Moral development<br>-Moral decision making<br>-Choice behavior<br>-Agency | -Palliative care<br>-End-of-life care<br>-Terminal care<br>-Palliative treatment<br>-Supportive care<br>-Palliative therapy<br>-Hospice care<br>-Hospices<br>-Palliation<br>-Comfort care<br>-Patient comfort | -Nursing<br>-Nurses<br>-Registered nurses<br>-Social work<br>-Social workers<br>-Social service<br>-Nurse attitudes<br>-Social work attitudes<br>-Nursing Care |

**Search strategy in the selected database: 1 -** CINAHL (EBSCOhost)

| | | |
|---|---|---|
| **Concept 1: Search equation with MeSH (S1)** | | (MH "Professional Identity") OR (MH "Professionalism") OR (MH "Self Concept+") OR (MH "Professional Image") OR (MH "Ethics, Professional+") OR (MH "Professional Competence") OR (MH "Professional Role") OR (MH "Nursing Role") |
| **Concept 1: Search equation with keywords (S2)** | | (professional* N2 (identity OR education OR formation OR training OR "self concept" OR value* OR image* OR comportement* OR conduct* OR ethic* OR dignity OR competence)) OR Professionali* OR (identity N2 (formation OR construction)) OR ((nurs* OR "social work*") N2 (identity OR ethic*)) OR (ethical N2 (identity OR comportment* OR practice* OR value*)) OR ((professional OR nurs* OR "social worker*") N2 role) |
| **Concept 1: Mixed equation (S3)** | **OR** | **(S1 OR S2)** |
| **Concept 2: Search equation with MeSH (S4)** | | (MH "Morals+") OR (MH "Decision Making, Ethical") |
| **Concept 2: Search equation with keywords (S5)** | | (moral N2 (agent* OR identity OR philosophy OR psychology OR conflict* OR judgement* OR value* OR decision* OR development)) OR agency OR "choice behavior" |
| **Concept 2: Mixed equation (S6)** | **OR** | **(S4 OR S5)** |
| **Concept 3: Search equation with MeSH (S7)** | | (MH "Terminal Care+") OR (MH "Hospice and Palliative Nursing") OR (MH "Hospices") OR (MH "Hospice Patients") |
| **Concept 3: Search equation with keywords (S8)** | | palliat* OR hospice* OR "end of life" OR "confort care" OR "patient comfort" OR ((Terminal OR Supportive) N2 (Care OR Treatment OR Therapy)) |
| **Concept 3: Mixed equation (S9)** | **OR** | **(S7 OR S8)** |
| **Concept 4: Search equation with MeSH (S10)** | | (MH "Nurses+") OR (MH "Nursing Practice+") OR (MH "Social Work+") OR (MH "Social Workers") OR (MH "Social Work Service") OR (MH "Social Work Practice") OR (MH "Nurse Attitudes") OR (MH "Social Worker Attitudes") OR (MH "Nursing Care+") |
| **Concept 4: Search equation with keywords (S11)** | | Nurs* OR (Social N2 (work* OR service*)) |
| **Concept 4: Mixed equation (S12)** | **OR** | **(S10 OR S11)** |
| **Final equation (S13)** | **AND** | **((S3 OR S6) AND S9 AND S12)** |

**Search strategy in the selected database: 2 -** MEDLINE (Ovid)

| | | |
|---|---|---|
| **Concept 1: Search equation with MeSH (S1)** | | Professional Role/ OR Nurse's Role/ OR Professional Competence/ OR Self Concept/ OR exp Ethics, Professional/ |
| **Concept 1: Search equation with keywords (S2)** | | (professional* adj2 (identity OR education OR formation OR training OR "self concept" OR value* OR image* OR comportement* OR conduct* OR ethic* OR dignity OR competence)) OR Professionali* OR (identity adj2 (formation OR construction)) OR ((nurs* OR "social work*") adj2 (identity OR ethic*)) OR (ethical adj2 (identity OR comportment* OR practice* OR value*)) OR ((professional OR nurs* OR "social worker*") adj2 role) |
| **Concept 1: Mixed equation (S3)** | **OR** | **(S1 OR S2)** |
| **Concept 2: Search equation with MeSH (S4)** | | exp Morals/ OR exp Decision Making/ |
| **Concept 2: Search equation with keywords (S5)** | | (moral adj2 (agent* OR identity OR philosophy OR psychology OR conflict* OR judgement* OR value* OR decision* OR development)) OR agency OR "choice behavior" |
| **Concept 2: Mixed equation (S6)** | **OR** | **(S4 OR S5)** |
| **Concept 3: Search equation with MeSH (S7)** | | Palliative Care/ OR exp Terminal Care/ OR Hospices/ OR Patient Comfort/ |
| **Concept 3: Search equation with keywords (S8)** | | palliat* OR hospice* OR "end of life" OR "confort care" OR "patient comfort" OR ((Terminal OR Supportive) adj2 (Care OR Treatment OR Therapy)) |
| **Concept 3: Mixed equation (S9)** | **OR** | **(S7 OR S8)** |
| **Concept 4: Search equation with MeSH (S10)** | | exp Nursing/ OR exp Nurses/ OR exp Nursing Care/ OR exp Social Work/ OR Social Workers/ |
| **Concept 4: Search equation with keywords (S11)** | | Nurs* OR (Social adj2 (work* OR service*)) |
| **Concept 4: Mixed equation (S12)** | **OR** | **(S10 OR S11)** |
| **Final equation (S13)** | **AND** | **((S3 OR S6) AND S9 AND S12)** |

**Search strategy in the selected database: 3 -** Embase (Ovid)

| | | |
|---|---|---|
| **Concept 1: Search equation with MeSH (S1)** | | self concept/ OR professional image/ OR professional competence/ OR professionalism/ OR vocational education/ OR professional ethics/ |
| **Concept 1: Search equation with keywords (S2)** | | (professional* adj2 (identity OR education OR formation OR training OR “self concept” OR value* OR image* OR comportement* OR conduct* OR ethic* OR dignity OR competence)) OR Professionali* OR (identity adj2 (formation OR construction)) OR ((nurs* OR "social work*") adj2 (identity OR ethic*)) OR (ethical adj2 (identity OR comportment* OR practice* OR value*)) OR ((professional OR nurs* OR "social worker*") adj2 role) |
| **Concept 1: Mixed equation (S3)** | **OR** | **(S1 OR S2)** |
| **Concept 2: Search equation with MeSH (S4)** | | ethics/ OR medical ethics/ OR ethical decision making/ OR exp morality/ |
| **Concept 2: Search equation with keywords (S5)** | | (moral adj2 (agent* OR identity OR philosophy OR psychology OR conflict* OR judgement* OR value* OR decision* OR development)) OR agency OR "choice behavior" |
| **Concept 2: Mixed equation (S6)** | **OR** | **(S4 OR S5)** |
| **Concept 3: Search equation with MeSH (S7)** | | exp palliative therapy/ OR exp terminal care/ OR hospice/ OR patient comfort/ |
| **Concept 3: Search equation with keywords (S8)** | | palliat* OR hospice* OR "end of life" OR "confort care" OR "patient comfort" OR ((Terminal OR Supportive) adj2 (Care OR Treatment OR Therapy)) |
| **Concept 3: Mixed equation (S9)** | **OR** | **(S7 OR S8)** |
| **Concept 4: Search equation with MeSH (S10)** | | exp nursing/ OR exp nurse/ OR nurse attitude/ OR exp nursing care/ OR social work/ OR social work practice/ OR social worker/ OR social worker attitude/ |
| **Concept 4: Search equation with keywords (S11)** | | Nurs* OR (Social adj2 (work* OR service*)) |
| **Concept 4: Mixed equation (S12)** | **OR** | **(S10 OR S11)** |
| **Final equation (S13)** | **AND** | **((S3 OR S6) AND S9 AND S12)** |

**Search strategy in the selected database: 4 -** PsycINFO (Ovid)

| | | |
|---|---|---|
| **Concept 1: Search equation with MeSH (S1)** | | professional identity/ OR identity formation/ OR exp personnel training/ OR exp professional competence/ OR self-concept/ OR professional ethics/ OR professional role/ |
| **Concept 1: Search equation with keywords (S2)** | | (professional* ADJ/2 (identity OR education OR formation OR training OR “self concept” OR value* OR image* OR comportement* OR conduct* OR ethic* OR dignity OR competence)) OR Professionali* OR (identity ADJ/2 (formation OR construction)) OR ((nurs* OR "social work*") ADJ/2 (identity OR ethic*)) OR (ethical ADJ/2 (identity OR comportment* OR practice* OR value*)) OR ((professional OR nurs* OR "social worker*") ADJ/2 role) |
| **Concept 1: Mixed equation (S3)** | **OR** | **(S1 OR S2)** |
| **Concept 2: Search equation with MeSH (S4)** | | moral development/ OR agency/ OR morality/ OR choice behavior/ OR decision making/ |
| **Concept 2: Search equation with keywords (S5)** | | (moral ADJ/2 (agent* OR identity OR philosophy OR psychology OR conflict* OR judgement* OR value* OR decision* OR development)) OR agency OR "choice behavior" |
| **Concept 2: Mixed equation (S6)** | **OR** | **(S4 OR S5)** |
| **Concept 3: Search equation with MeSH (S7)** | | exp palliative care/ OR hospice/ |
| **Concept 3: Search equation with keywords (S8)** | | palliat* OR hospice* OR "end of life" OR "confort care" OR "patient comfort" OR ((Terminal OR Supportive) ADJ/2 (Care OR Treatment OR Therapy)) |
| **Concept 3: Mixed equation (S9)** | **OR** | **(S7 OR S8)** |
| **Concept 4: Search equation with MeSH (S10)** | | exp nurses/ OR nursing/ OR exp social casework/ OR exp social workers/ OR exp social services/ |
| **Concept 4: Search equation with keywords (S11)** | | Nurs* OR (Social ADJ/2 (work* OR service*)) |
| **Concept 4: Mixed equation (S12)** | **OR** | **(S10 OR S11)** |
| **Final equation (S13)** | **AND** | **((S3 OR S6) AND S9 AND S12)** |

**Search strategy in the selected database: 5 –** Social Work Abstracts (EBSCO)
**6 –** Social Sciences Abstracts (EBSCO)

| | | |
|---|---|---|
| **Concept 1: Search equation with keywords (S1)** | | (professional* N2 (identity OR education OR formation OR training OR “self concept” OR value* OR image* OR comportement* OR conduct* OR ethic* OR dignity OR competence)) OR Professionali* OR (identity N2 (formation OR construction)) OR ((nurs* OR "social work*") N2 (identity OR ethic*)) OR (ethical N2 (identity OR comportment* OR practice* OR value*)) OR ((professional OR nurs* OR "social worker*") N2 role) |
| | | |
| **Concept 2: Search equation with keywords (S2)** | | (moral N2 (agent* OR identity OR philosophy OR psychology OR conflict* OR judgement* OR value* OR decision* OR development)) OR agency OR "choice behavior" |
| | | |
| **Concept 3: Search equation with keywords (S3)** | | palliat* OR hospice* OR "end of life" OR "confort care" OR "patient comfort" OR ((Terminal OR Supportive) N2 (Care OR Treatment OR Therapy)) |
| | | |
| **Concept 4: Search equation with keywords (S4)** | | Nurs* OR (Social N2 (work* OR service*)) |
| | | |
| **Final equation (S13)** | **AND** | **((S1 OR S2) AND S3 AND S4)** |

**Search strategy in the selected database: 7 –** Social Services Abstracts (ProQuest)
**8 –** Sociological Abstracts (ProQuest)

| | | |
|---|---|---|
| **Concept 1: Search equation with MeSH (S1)** | | SU.EXACT("Professional Identity") OR SU.EXACT("Professionalization") OR SU.EXACT("Professionalism") OR SU.EXACT("Professional Training") OR SU.EXACT("Self Concept") OR SU.EXACT("Professional Ethics") OR SU.EXACT("Occupational Roles") |
| **Concept 1: Search equation with keywords (S2)** | | (professional* NEAR/2 (identity OR education OR formation OR training OR "self concept" OR value* OR image* OR comportement* OR conduct* OR ethic* OR dignity OR competence)) OR Professionali* OR (identity NEAR/2 (formation OR construction)) OR ((nurs* OR "social work*") NEAR/2 (identity OR ethic*)) OR (ethical NEAR/2 (identity OR comportment* OR practice* OR value*)) OR ((professional OR nurs* OR "social worker*") NEAR/2 role) |
| **Concept 1: Mixed equation (S3)** | **OR** | **(S1 OR S2)** |
| **Concept 2: Search equation with MeSH (S4)** | | SU.EXACT("Moral Development") OR SU.EXACT("Moral Judgment") OR SU.EXACT("Morality") |
| **Concept 2: Search equation with keywords (S5)** | | (moral NEAR/2 (agent* OR identity OR philosophy OR psychology OR conflict* OR judgement* OR value* OR decision* OR development)) OR agency OR "choice behavior" |
| **Concept 2: Mixed equation (S6)** | **OR** | **(S4 OR S5)** |
| **Concept 3: Search equation with MeSH (S7)** | | SU.EXACT("Hospices") OR SU.EXACT("Palliative Care") |
| **Concept 3: Search equation with keywords (S8)** | | palliat* OR hospice* OR "end of life" OR "confort care" OR "patient comfort" OR ((Terminal OR Supportive) NEAR/2 (Care OR Treatment OR Therapy)) |
| **Concept 3: Mixed equation (S9)** | **OR** | **(S7 OR S8)** |
| **Concept 4: Search equation with MeSH (S10)** | | SU.EXACT("Nurses") OR SU.EXACT("Social Workers") OR SU.EXACT.EXPLODE("Social Work") OR SU.EXACT.EXPLODE("Social Services") |
| **Concept 4: Search equation with keywords (S11)** | | Nurs* OR (Social NEAR/2 (work* OR service*)) |
| **Concept 4: Mixed equation (S12)** | **OR** | **(S10 OR S11)** |
| **Final equation (S13)** | **AND** | **((S3 OR S6) AND S9 AND S12)** |

**Search strategy in the selected database: 9 –** Dissertations and Theses Global (ProQuest)

| | | |
|---|---|---|
| **Concept 1: Search equation with MeSH (S1)** | | EXACT("professional education" OR "professional ethics" OR "identity formation") |
| **Concept 1: Search equation with keywords (S2)** | | (professional* NEAR/2 (identity OR education OR formation OR training OR “self concept” OR value* OR image* OR comportement* OR conduct* OR ethic* OR dignity OR competence)) OR Professionali* OR (identity NEAR/2 (formation OR construction)) OR ((nurs* OR “social work*”) NEAR/2 (identity OR ethic*)) OR (ethical NEAR/2 (identity OR comportment* OR practice* OR value*)) OR ((professional OR nurs* OR “social worker*”) NEAR/2 role) |
| **Concept 1: Mixed equation (S3)** | **OR** | **(S1 OR S2)** |
| **Concept 2: Search equation with MeSH (S4)** | | EXACT("morality") |
| **Concept 2: Search equation with keywords (S5)** | | (moral NEAR/2 (agent* OR identity OR philosophy OR psychology OR conflict* OR judgement* OR value* OR decision* OR development)) OR agency OR "choice behavior" |
| **Concept 2: Mixed equation (S6)** | **OR** | **(S4 OR S5)** |
| **Concept 3: Search equation with MeSH (S7)** | | EXACT("palliative care" OR "hospice care") |
| **Concept 3: Search equation with keywords (S8)** | | palliat* OR hospice* OR "end of life" OR "confort care" OR "patient comfort" OR ((Terminal OR Supportive) NEAR/2 (Care OR Treatment OR Therapy)) |
| **Concept 3: Mixed equation (S9)** | **OR** | **(S7 OR S8)** |
| **Concept 4: Search equation with MeSH (S10)** | | EXACT("nurses" OR "nursing" OR "social workers" OR "social work") |
| **Concept 4: Search equation with keywords (S11)** | | Nurs* OR (Social NEAR/2 (work* OR service*)) |
| **Concept 4: Mixed equation (S12)** | **OR** | **(S10 OR S11)** |
| **Final equation (S13)** | **AND** | **((S3 OR S6) AND S9 AND S12)** |

**Search strategy in the selected database: 10 –** Web of Science (Clarivate)

| | | |
|---|---|---|
| **Concept 1: Search equation with keywords (S1)** | | (professional* NEAR/2 (identity OR education OR formation OR training OR “self concept” OR value* OR image* OR comportement* OR conduct* OR ethic* OR dignity OR competence)) OR Professionali* OR (identity NEAR/2 (formation OR construction)) OR ((nurs* OR “social work*”) NEAR/2 (identity OR ethic*)) OR (ethical NEAR/2 (identity OR comportment* OR practice* OR value*)) OR ((professional OR nurs* OR “social worker*”) NEAR/2 role) |
| | | |
| **Concept 2: Search equation with keywords (S2)** | | (moral NEAR/2 (agent* OR identity OR philosophy OR psychology OR conflict* OR judgement* OR value* OR decision* OR development)) OR agency OR "choice behavior" |
| | | |
| **Concept 3: Search equation with keywords (S3)** | | palliat* OR hospice* OR "end of life" OR "confort care" OR "patient comfort" OR ((Terminal OR Supportive) NEAR/2 (Care OR Treatment OR Therapy)) |
| | | |
| **Concept 4: Search equation with keywords (S4)** | | Nurs* OR (Social NEAR/2 (work* OR service*)) |
| | | |
| **Final equation (S13)** | **AND** | **((S1 OR S2) AND S3 AND S4)** |

**Search strategy in the selected database: 11 –** JBI EBP Database (Ovid)

| | | |
|---|---|---|
| **Concept 1: Search equation with keywords (S1)** | | (professional* adj2 (identity OR education OR formation OR training OR “self concept” OR value* OR image* OR comportement* OR conduct* OR ethic* OR dignity OR competence)) OR Professionali* OR (identity adj2 (formation OR construction)) OR ((nurs* OR "social work*") adj2 (identity OR ethic*)) OR (ethical adj2 (identity OR comportment* OR practice* OR value*)) OR ((professional OR nurs* OR "social worker*") adj2 role) |
| | | |
| **Concept 2: Search equation with keywords (S2)** | | (moral adj2 (agent* OR identity OR philosophy OR psychology OR conflict* OR judgement* OR value* OR decision* OR development)) OR agency OR "choice behavior" |
| | | |
| **Concept 3: Search equation with keywords (S3)** | | palliat* OR hospice* OR "end of life" OR "confort care" OR "patient comfort" OR ((Terminal OR Supportive) adj2 (Care OR Treatment OR Therapy)) |
| | | |
| **Concept 4: Search equation with keywords (S4)** | | Nurs* OR (Social adj2 (work* OR service*)) |
| | | |
| **Final equation (S13)** | **AND** | **((S1 OR S2) AND S3 AND S4)** |

**Search strategy in the selected database: 12 –** EBM Reviews - Cochrane Database of Systematic Reviews (Ovid)

| | | |
|---|---|---|
| **Concept 1: Search equation with MeSH (S1)** | | education, professional/ OR education, nursing/ OR self concept/ OR exp ethics, professional/ OR exp professional role/ OR professional competence/ |
| **Concept 1: Search equation with keywords (S2)** | | (Professional* adj2 (identity OR education OR formation OR training OR "self concept" OR value* OR image OR comportement* OR conduct* OR ethic* OR dignity)) OR Professionali* OR "identity formation" OR ((nurs* OR "social work*") adj2 identity) OR (ethical adj2 (identity OR comportment* OR practice* OR value*)) |
| **Concept 1: Mixed equation (S3)** | **OR** | **(S1 OR S2)** |
| **Concept 2: Search equation with MeSH (S4)** | | exp morals/ OR decision making/ OR choice behavior/ |
| **Concept 2: Search equation with keywords (S5)** | | Moral adj2 (agency OR agent OR identity OR philosophy OR psychology OR conflict* OR judgement* OR value*) |
| **Concept 2: Mixed equation (S6)** | **OR** | **(S4 OR S5)** |
| **Concept 3: Search equation with MeSH (S7)** | | palliative care/ OR exp terminal care/ OR hospices/ |
| **Concept 3: Search equation with keywords (S8)** | | Palliat* OR hospice* OR "End of life" OR ((Terminal OR Supportive) adj2 (Care OR Treatment OR Therapy)) |
| **Concept 3: Mixed equation (S9)** | **OR** | **(S7 OR S8)** |
| **Concept 4: Search equation with MeSH (S10)** | | exp nurses/ OR exp nursing staff/ OR exp nursing/ OR exp social work/ |
| **Concept 4: Search equation with keywords (S11)** | | Nurs* OR (Social adj2 (work* OR service*)) |
| **Concept 4: Mixed equation (S12)** | **OR** | **(S10 OR S11)** |
| **Final equation (S13)** | **AND** | **((S3 OR S6) AND S9 AND S12)** |

Supplementary Material 2: Characteristics of the 38 included articles

| Reference | Type of article, discipline, and country | Objective | Methods | Results pertaining to moral agency | Results pertaining to professional identity | Reflections in relation to the concepts | Quality Appraisal |
|---|---|---|---|---|---|---|---|
| 1. Albanesi | Literature | Provide an overview | Scoping review | Moral distress occurs when | The cessation of the | The study would seem | High |

| | | | | | | | |
|---|---|---|---|---|---|---|---|
| et al. (2020) | review Nursing Italy | of nurses' extensive and multifaceted involvement in artificial nutrition and hydration (ANH) at the end of life and identify key underlying concepts to inform practice development | | there is an imbalance between "personal-self", do what is good according to moral judgment, and the "professional-self", do what is necessary for the patient, factors posing barriers: bad communication, ineffective teamwork, lack of structure in personalized care plans, insufficient knowledge of ethical principles. | Artificial Nutition and Hydratation generates a feeling in nurses to "leave patients without care", which goes against their vision of the professional mission; care techniques grounded in their self-representation as a professional. | to suggest a link between moral agency and professional identity insofar as the moral judgment of what is "good" can in certain circumstances go against the professional mission perceived by nurses, the way in which they conceive their professional role (professional identity). | (JBI) |
| 2. Bellens et al. (2020) | Empirical study Nursing Belgium | Explore the experience of nurses in the Flemish region, working in hospital or in home care, in connection with their involvement with patients asking for euthanasia | Grounded theory | Enablers: Access to euthanasia allows nurses to contribute to a "good death" in dignity = professional development. Communication between patient-family-nurses. Barriers: Pressure to act when patient is in “mental distress”, lack of time, lack of teamwork, value conflicts (“unnatural” death), feeling of not having the capacity to accompany the patient and family. | | The concept of ethical responsibility in nurses’ understanding of their role was only marginally present in the interviews. This suggests that nurses may act less as moral agents and more as advocates for patients' wishes—regardless of the nature or ethical implications of those wishes (reflecting a utilitarian stance). This raises the idea that acting as a patient advocate does not necessarily equate to exercising moral agency. | High (MMAT) |
| 3. Beuthin et al. (2018) | Empirical study Nursing Canada | Understand the range of nurses’ experience in providing care for someone choosing MAiD, whether directly aiding, providing supportive care, or declining to | Narrative Inquiry | | Enablers: Participating in MAiD reinforced a sense of their role in providing comfort care, mitigating suffering. | The quotes in the "Strongly Oppose" section (p. 515) suggest that MAiD may challenge individuals’ personal identity, but not necessarily their professional identity. | High (MMAT) |

| | | | | | | | |
|---|---|---|---|---|---|---|---|
| | | participate. | | | | | |
| 4. Bradshaw et al. (2022) | Empirical study<br>Nursing (50%), and other healthcare professionals<br>United Kingdom | Understand the multinational response of specialist palliative and hospice care services to the COVID-19 pandemic | Descriptive qualitative multiple case study | Infection control policies and procedures were the most frequently cited constraints preventing nurses from practicing in accordance with their professional values. These restrictions triggered moral distress by impeding their ability to deliver care aligned with those values—particularly in caring for dying patients, honoring patient wishes (such as leaving the hospital), and establishing meaningful relationships with patients and families. Measures like visitation limits and PPE requirements further acted as barriers to communication and connection. | | | High (MMAT) |
| 5. Carnevale (2020) | Theoretical article<br>Nursing<br>Canada | Outline strategies that could be used to help diminish moral distress, drawing on the available literature | Theoretical exploration | Enablers: Addressing and reducing barriers is essential to resolving moral distress (p. 457). Ethics education and the creation of structured discussion forums contribute to the development of moral agency (p. 458). Peer support encourages open and empathetic ethical dialogue (p. 458), while moral resilience reinforces ethical practice (p. 457). Moral courage, in turn, is a concrete expression of moral agency (p. 459). Barrier (and potential enabler). Divergent moral | | Moral agency may not solely rely on the removal of barriers, but rather on the strengthening of enablers—that is, fostering the supportive conditions that empower nurses to act as moral agents. The core issue may lie less in the presence of obstacles than in the absence of essential conditions that allow moral agency to emerge and be sustained. | High (JBI) |

| | | | | | | | |
|---|---|---|---|---|---|---|---|
| | | | | perspectives among colleagues can be a source of distress, but they may also create opportunities for meaningful ethical dialogue (p. 458). | | | |
| 6. Dorman and Bouchal (2020) | Literature review<br>Nursing<br>Canada | Develop a simultaneous, evolutionary concept analysis of moral distress and moral uncertainty in the context of MAiD | Concept analysis according to Rodger's and Haase | Emotional reactions, silence, and limited team or policy support create uncertainty (p. 323–324). External limits—policy, budget, time, curative culture—restrict nurses (p. 325). | Moral distress can hinder identity (p. 325), but also foster growth through reflection, resilience, and self-knowledge (p. 325). | It is interesting to see the range of possible decisions (not always yes or no, there are times when participants do not know what to do). | Average (JBI) |
| 7. Dressler et al. (2021) | Empirical study<br>Mixed (nursing, social work), including managers, administrative personnel, and other healthcare professionals<br>United States | Characterize hospice staff practices and perspectives on discussing end-of-life care preferences with patients, families | Qualitative, descriptive multicenter study | Participants in this study reported challenges in end-of-life care discussions within hospice, specifically concerning patient/family preferences for intensive treatment and full code status. This created tension and ethical dilemmas for caregivers striving to balance respecting wishes with preventing harm and alleviating suffering, revealing conflict within hospice philosophy. | | | Average (MMAT) |
| 8. Elmore et al. (2018) | Literature review<br>Nursing<br>Canada | Understand, the nursing experience of caring for patients who request assisted death | Qualitative meta-synthesis | Enablers: Moral significance is attached to the interactions they have with patients and families, and the opportunity to have these interactions greatly influences the experience of the nurse (p.965).<br>Training/Education; organizational support; communication/collaboratio n.<br>Barriers: Pressures/external expectations; | | | High (JBI) |

| | | | | | | | |
|---|---|---|---|---|---|---|---|
| | | | | conflicts/values; lack of resources. | | | |
| 9. Falco-Pegueroles et al. (2023) | Empirical study Nursing, including physicians Spain | Explore in depth the sources of ethical conflict of ICU nurses and physicians and their decision-making processes | Descriptive phenomenological study | They requested that the hospital's management grant permission for a family member to accompany the patient at the end of life. However, in Italy this proved impossible as the restrictions on hospital visits remained in place during both the first and second waves (p. 5191). | | | High (MMAT) |
| 10. Fantus et al. (2017) | Theoretical article Social work Canada | Clarify how moral distress manifests in hospital social work | Theoretical exploration | Budgets, staff shortages, and workload hinder social workers' moral action (p. 2280). Ethics education supports moral efficacy and courage to ease distress (p. 2285). | Moral distress affects identity and agency, shaped by internal and systemic factors (p. 2276). It may harm work climate and communication, or foster reflection, empathy, and moral sensitivity (p. 2276). | Very focused on moral distress. Their definition of moral courage (p. 2285) resembles ours as for moral agency. | High (JBI) |
| 11. Fortier (2018) | Empirical study Nursing Canada | Explore moral agency and moral distress in novice and expert nurses, focusing on the influence of organizational and bureaucratic factors, and comparing their moral agency | Qualitative interpretive approach, based on De Groot | Time constraints reduce emotional care, foster task-orientation, and hinder moral agency (p. 48, 51, 62). Novice nurses feel voiceless and fear retaliation (p. 68), while gaining confidence and senior support fosters moral agency (p. 70–72). Feeling unheard blocks agency (p. 73). Reflecting on past inaction aids growth (p. 87); moral communities can support moral agency (p. 99). | Inability to develop a relationship and to advocate for patients due to the missing time. Moral agency and professional identity intertwine according to the philosophy of existentialism. | | High (MMAT) |
| 12. Funk et | Empirical | Explore how paid care | Interpretive | Staff shortages and | Healthcare staff | The text examines how | High |

| | | | | | | | |
|---|---|---|---|---|---|---|---|
| al. (2017) | study Nursing Canada | providers understand and interpret grief when working with dying patients and families, and the emotional labor they engage in as they manage grief in the context of their work | inquiry | overwhelming workloads hindered providers from easing suffering or supporting dignified deaths, while insufficient processing time between patient losses compromised their ability to support subsequent families. | face contradictory pressures: expected to care while suppressing emotions in cultures where vulnerability equals weakness. Support networks and environments that normalize emotional expression are key solutions. | barriers to professional identity development conflict with caregivers' ability to facilitate 'good deaths' as moral agents. | (MMAT) |
| 13. Gebrehea t and Teame (2021) | Literature review Nursing Ethiopia | Identify the main ethical challenges faced by nurses during the COVID-19 pandemic | Integrative review | Key ethical challenges for nurses during the pandemic included inadequate protection and PPE, leading to questions about their duty; the difficult and often unclear decisions regarding fair allocation of scarce medical resources; insufficient responses to urgent needs; and being required to work beyond their job descriptions, potentially causing moral distress and compromising the quality of care for patients and communities. | | This article highlights ethical nursing issues, which are revealed /associate with barriers to moral agency. | High (JBI) |
| 14. Glasdam et al. (2020) | Empirical study Nursing Sweden | Explore nursing practice in specialized palliative homecare, and how it is influenced by organizational and culture structures | Exploratory qualitative study | Nurses could only meet patients' psychosocial needs during rare schedule gaps, contradicting their care ideals. Some found fulfillment by deliberately working outside system constraints. | | | High (MMAT) |
| 15. Hold (2017) | Empirical study Nursing United States | Explore how experienced hospice nurses resolve day-to-day ethical dilemmas | Qualitative narrative approach | Enablers for nurses in facilitating a good death included educating families and patients about symptom | Nurses expressed frustration due to stakeholders hindering their | This article has interesting points between moral agency and professional | High (MMAT) |

| | | | | | | | |
|---|---|---|---|---|---|---|---|
| | | during end-of-life care | | management and the disease process, building strong relationships, and utilizing ethical knowledge derived from experience, intuition, and formal training to find ethical solutions. Nurses' own values and moral insights, along with intuition, also guided their decision-making. Barriers to achieving this goal involved frustration stemming from interference by stakeholders (hospice companies, providers, or families) with conflicting objectives. | ability to fulfill professional responsibilities in achieving positive patient outcomes, with conflicting objectives from hospice companies, physicians, or other healthcare staff cited as impediments. | identity. | |
| 16. Jerpseth et al. (2017) | Empirical study Nursing Norway | Elucidate how nurses experience their own role and care in end-of-life decision-making processes regarding severely ill COPD patients | Descriptive qualitative study | A medical culture prioritizing treatment over comfort and ambiguous professional roles left nurses feeling powerless to act according to their caring values and perceived best interests of severely ill COPD patients, particularly in decision-making and care for those with limited future treatment options. | | Subordination by the medical profession might be related to moral agency. | Average (MMAT) |
| 17. Ko et al. (2018) | Empirical study Nursing Taiwan | Reconstruct a grounded theory model of moral distress by:<br>(a) understanding Taiwanese nurses' moral values;<br>(b) structuring values, barriers, and actions into a clear model;<br>(c) identifying nurses' moral actions based on the known effects | Grounded theory | Enablers for moral action included respecting patient decisions and providing necessary information, cooperative nurses, supportive managers, open-minded physicians, fair staffing, and self-confidence promoting moral efficacy. | | Although the model primarily focuses on moral distress rather than moral agency, it nonetheless offers insights into how to support moral action aligned with nurses' values, that is, their moral judgment of what is "good." In this way, it becomes possible to identify | High (MMAT) |

| | | | | | | | |
|---|---|---|---|---|---|---|---|
| | | of moral distress | | | | enablers that support the development and expression of moral agency. | |
| 18. Kopchek (2020) | Empirical study Nursing Canada | Explore the ethical decision-making experiences of Ontario palliative care nurses related to MAiD | Interpretive description | A lack of knowledge about MAiD and foundational ethical principles undermined palliative care nurses' ability to make informed ethical decisions, thereby hindering their personal vision of what constitutes good care. | | | High (MMAT) |
| 19. Laugerat et al. (2023) | Theoretical article Nursing France | Expose experiences with implemented actions in extra-hospital settings during the isolation period | Practice narrative | The pandemic challenged caregivers' identities and abilities, leaving them feeling unsupported and uncertain despite recognizing their vital roles. Facilitating the expression of their experiences and doubts helped them develop reflective practices and collaboratively adapt care strategies to the unprecedented challenges. | They enabled healthcare providers—who were experiencing a crisis of identity and role—to shift from individual distress toward an acceptance of uncertainty, fostering the emergence of collective care practices and responses better adapted to the situation. | | Average (JBI) |
| 20. Lim and Kim (2021) | Empirical study Nursing Korea | Identify Korean nurses' ethical decision-making process based on their moral sensitivity to end-of-life patients | Quantitative cross-sectional, descriptive Survey | 10+ years of clinical experience strengthens moral agency. Ethics education (especially 10+ hours), department type, job satisfaction, and conflict levels collectively predict ethical decision-making ability, explaining nearly half its variance. | | The study provides empirical evidence that ethics education strengthens nurses' moral agency, a previously unverified recommendation. However, statistical methods may inadequately reveal the complete ethical decision-making process. | High (MMAT) |

| | | | | | | | |
|---|---|---|---|---|---|---|---|
| 21. Lokker et al. (2018) | Empirical study<br>Nursing<br>The Netherlands | Explore nurses' reports on the practice of palliative sedation focusing on their experiences of pressure, di- lemmas and morally distressing situations | Exploratory qualitative study | Hierarchical differences and pressure to act against their judgment hindered nurses' involvement in palliative sedation decisions. Adequate clinical and ethical knowledge was identified as crucial for nurses to effectively participate and advocate for patient well-being. | A significant barrier to nurses' moral agency might also be related to professional identity, which is the devaluation of their observations and experience by physicians ("You're just a nurse"), undermining their professional judgment and scope of practice. | The hierarchical difference between physicians and nurses can impede nurses' moral agency, making them feel powerless in decisions. This can lead nurses to internalize this powerlessness, diminishing their professional identity by thinking of themselves as "only a nurse." The question is how to prevent this initial lack of agency from negatively impacting their sense of professional identity. | Low (MMAT) |
| 22. Ma et al. (2020) | Empirical study<br>Nursing<br>Canada | Explore a diversity of experiences that nurses consider "meaningful" in their practice, with an explicit focus on the moral dimensions of these experiences | Interpretive description | A key barrier is physicians holding exclusive decision-making power, limiting nurses' involvement. Enablers include reflection fostering moral growth, experience building moral agency, knowing the patient enabling ethical care, critical resilience driving systemic change, and moral sensitivity supporting ethical reasoning. | | | High (MMAT) |
| 23. McMillan et al. (2021) | Empirical study<br>Nursing<br>Canada | Explore the ethical issues encountered by palliative care nurses in the context of COVID-19 and to examine how they respond to and navigate these challenges in their clinical practice | Interpretive description | COVID-19 policies, such as visit restrictions, created a barrier by conflicting with nurses' ethical duty to ensure a good death, causing them ethical dissonance when enforcing these rules. Enablers included nurses advocating and sometimes breaking | | Enablers and barriers are very specific to COVID-19. | High (MMAT) |

| | | | | | | | |
|---|---|---|---|---|---|---|---|
| | | | | rules to uphold palliative values, and supportive relationships with clinical directors allowing for ethical flexibility. | | | |
| 24. Meeker and White (2021) | Empirical study Nursing United States | Examine the experiences of registered nurses in acute care settings as they worked with patients and families to facilitate transition to comfort-focused care | Qualitative research guided by constructivist grounded theory | Strong, trusting nurse-patient relationships were foundational for facilitating comfort-focused care, enabling nurses to honor patient autonomy and provide support. Achieving consensus among providers, patients, and families through nurses' mediation was also key, guided by shared moral principles. | Over time, one participant reported a shift towards being more open about sharing her own values to guide end-of-life decision-making with her family. | | High (MMAT) |
| 25. Mowat et al. (2023) | Empirical study Nursing New Zealand | Explore the moral emotions that frontline nurses navigated in endeavoring to ensure a ‘good death’ for hospital patients and care home residents during the first wave of COVID-19 | Theory-driven qualitative study | Frontline workers collaborated to make morally sound decisions, balancing new rules with their conviction that dying individuals should be with family. Despite moral distress, their teamwork and adaptability allowed them to optimize care in challenging pandemic circumstances, a source of professional pride. | | | High (MMAT) |
| 26. O'Mathúna et al. (2023) | Empirical study Nursing United States | Describe and clarify the experiences of frontline U.S. nurses during COVID-19 that contained ethical dimensions | Grounded theory | Moral uncertainty arose from complex ethical dilemmas and lack of information. While the right action was sometimes clear, enacting it required moral courage, as nurses faced challenges like family resistance to comfort measures and stood firm on their ethical beliefs despite difficulties. | | | High (MMAT) |
| 27. Panozzo | Empirical | Explore experiences | Qualitative study | The presence of guards can | | The study is heavily | High |

| | | | | | | | |
|---|---|---|---|---|---|---|---|
| et al. (2020) | study Nursing, including other healthcare professionals Australia | and perspectives of health professionals regarding the provision of palliative and end-of-life care for hospitalized prisoner patients | | hinder the development of a therapeutic alliance with patients. A lack of understanding of the prison system can impede the provision of good palliative care. The context of incarceration, such as cell confinement, can restrict the administration of as-needed (PRN) medications. | | focused on the prison system, which may limit its transferability to other contexts; nonetheless, it offers highly valuable and thought-provoking insights. | (MMAT) |
| 28. Peter et al. (2018) | Empirical study Nursing Canada | Explore how nurses narrate their moral identity through their understanding of their work, and understand how these moral identities are developed and held in the social space that occupy | Grounded theory | | Nurses' moral identity is enabled by their core value of making a difference, positive patient interactions, a collective caring ethos, and societal expectations of a "good nurse," reinforced by their skills and the act of restoring patients' identities. Barriers include negative stereotypes, misrecognition hindering their moral actions, and the emotional toll of performing an inauthentic identity for patient approval. | A rare paper about enablers and barriers to the construction of professional identity among nurses. | High (MMAT) |
| 29. Prompahakul and Epstein (2020) | Literature review Nursing Thailand | Identify, describe, and synthesize previous primary studies on moral distress experienced by non-Western nurses | Integrative review | Barriers: Time pressure hinders quality care (p. 789). power imbalance limits nurses’ ability to advocate (p. 789), fear of retribution prevents ethical action (p. 790). Enablers: Some nurses defy | | | Average (JBI) |

| | | | | | | | |
|---|---|---|---|---|---|---|---|
| | | | | rules to protect patients (p. 790). | | | |
| 30. Rainer et al. (2018) | Literature review Nursing United States | Identify themes and gaps in what is known about handling ethical dilemmas in clinical nursing practice | Integrative review | Barriers: Staffing shortages forcing nurses to ration care and decide who receives necessary treatment often led to ethical dilemmas. Missed nursing care is also recognized as a significant threat to quality care and positive patient outcomes, with a clear link to ethical dilemmas in the workplace. Enablers: More ethically mature nurses, possessing ethical sensitivity (awareness of ethical situations), made better decisions in dilemmas. | | | High (JBI) |
| 31. Ramvi and Ueland (2019) | Empirical study Nursing Norway | Explore the ethical challenges that nurses encounter in their interactions with next of kin during end-of-life care | Critical qualitative approach | A barrier to nurses' moral agency is feeling unable to follow their values and having their expertise devalued, often due to protective next-of-kin dynamics hindering partnerships. An enabler for their confidence as moral agents is perceiving a positive relationship with the next-of-kin, where they feel seen as 'doing good.' | A barrier to nurses' professional identity is when the next-of-kin devalues their skills and role, perceiving their care as no better than what a family member could provide. This can reinforce a low social status and belittle the expertise required for quality professional care, leading to the feeling of being "only a nurse." | A rare and valuable contribution that explicitly articulates the connection between moral agency and professional identity. | High (MMAT) |
| 32. Reed et al. (2018) | Empirical study Nursing Australia | Develop a practice model for rural district nursing successful end-of-life advocacy care | Sequential mixed-methods study | Feeling supported by managers, friends, colleagues, other healthcare professionals increase self-confidence in the ability to | | Nurse agency – While not synonymous with moral agency, it closely resembles it, albeit without the | Average (MMAT) |

| | | | | | | | |
|---|---|---|---|---|---|---|---|
| | | | | assess situations and take action to promote end-of-life care (p. 749). Emotional intelligence is required to exercise moral agency, that is to understand the wishes of the dying person in order to make coherent decisions with them (p. 749). | | explicit ethical dimension. | |
| 33. Rodney (2017) | Theoretical article Nursing, but other disciplines briefly discussed (social work, medicine, psychology) Canada | Present an overview of moral distress by including existing research on this concept and discuss the ways of evolving our understanding of moral distress to meet the challenges of current and future practice | Theoretical exploration | Barriers (which thus lead to moral distress): lack of nursing staff, conflicts between clinicians, overload of work, challenges encountered in the decision at the end of life, efficiency pressure, poor working environment. | | | High (JBI) |
| 34. van den Bosch et al. (2023) | Empirical study Nursing The Netherlands | Identify moral challenges experienced by nurses and volunteers in palliative care | Qualitative hermeneutical design | Protocols hindering necessary safety measures (like bedrails without consent) and resource shortages preventing fulfillment of patient wishes caused moral dilemmas for caregivers, forcing them to balance patient well-being with rules and staff limitations, ultimately obstructing what they felt was good care and creating moral doubts. | | | High (MMAT) |
| 35. Variath et al. (2022) | Empirical study Nursing, including social work Canada | Explore Canadian healthcare providers' perspectives on providing MAiD to eligible patients in the absence of a contemporaneous final consent following their loss of decision- | Qualitative, incorporating a critical approach and concepts taken from feminist ethics | Anticipated barriers to moral agency in providing MAiD included feeling powerless against legal/familial challenges and resource limitations, concerns about family objections preventing MAiD fulfillment, and | | Illustrates the changes in the barriers and enablers to moral agency before the new legislation on MAiD. Allows us to understand that barriers and enablers in the context of our study are | High (MMAT) |

| | | | | | | | |
|---|---|---|---|---|---|---|---|
| | | making capacity | | organizational/legal constraints hindering the provision of a desired death (e.g., withholding MAiD if a confused patient resists). An anticipated enabler to minimize provider distress was the potential waiver of final consent, allowing them to honor a patient's previously expressed wish for MAID even if they lose capacity. | | dynamic over time. | |
| 36. Voultsos et al. (2023) | Empirical study Nursing Greece | Gain a deeper insight into nurses' perceptions and experiences of making conscientious objection to participating in nursing activities to be executed in situations involving end-of-life patients | Descriptive qualitative study | A major barrier to nurses' conscientious objection was perceived, power imbalance with physicians/managers, leading to feeling forced to comply with recommendations against their conscience. This was often internalized as physician authority being inherent to their profession. Fear of isolation, bullying, and gossip also prevented nurses from raising objections. Participants consistently expressed a need for more workplace support and legal protection for their right to conscientious objection without fear of job loss, highlighting the lack of clear legal and practice guidelines as a substantial barrier. | | | High (MMAT) |
| 37. Wright et al. (2021) | Empirical study Nursing Canada | Understand how palliative care nurses negotiate their moral identity in relation to MAiD | Qualitative research study with a feminist approach | | Barriers: Fear of expressing dissenting views, blurring of personal/profession | Explains several enablers and many constraints related to the professional nursing/ professional | High (MMAT) |

| | | | | | | | |
|---|---|---|---|---|---|---|---|
| | | | | | al values, ethical hesitation limiting therapeutic presence, and the disruptive personal reflection needed to reconcile MAiD with palliative values all hinder nurses' moral identity and agency.<br>Enablers: Nurses' identities and moral standing are reinforced through mutual recognition in relationships and the act of care. Reflecting on MAiD is a process of negotiating values and professional roles. | identity. | |
| 38. Wright et al. (2020) | Literature review<br>Nursing<br>Canada | Review existing nursing ethics literature about palliative sedation, and to analyze how nurses' moral identities are portrayed within this literature | Systematic review of normative literature | Moral identity work empowers moral agency. Deferring moral decisions to authority figures hinders it. Examining and reflecting on seemingly certain moral perspectives can lead to more informed and nuanced decision-making. | | Moral identity is distinct from professional identity, as it relates more to one’s internalized moral values than to one's role-based professional self-concept. | High (JBI) |

## References


Albanesi, B., Marchetti, A., D'Angelo, D., Capuzzo, M. T., Mastroianni, C., Artico, M., Lusignani, M., Piredda, M., & De Marinis, M. G. (2020). Exploring nurses' involvement in artificial nutrition and hydration at the end of life: A scoping review. *Journal of Parenteral and Enteral Nutrition*, *44*(7), 1220-1233. https://doi.org/10.1002/jpen.1772

Bellens, M., Debien, E., Claessens, F., Gastmans, C., & de Casterle, B. D. (2020). "It is still intense and not unambiguous." Nurses' experiences in the euthanasia care process 15 years after legalisation. *Journal of Clinical Nursing*, *29*(3-4), 492-502. https://doi.org/10.1111/jocn.15110

Beuthin, R., Bruce, A., & Scaia, M. (2018). Medical assistance in dying (MAiD): Canadian nurses' experiences. *Nursing Forum*, *53*(4), 511-520. https://doi.org/10.1111/nuf.12280

Bradshaw, A., Dunleavy, L., Garner, I., Preston, N., Bajwah, S., Cripps, R., Fraser, L. K., Maddocks, M., Hocaoglu, M., Murtagh, F. E. M., Oluyase, A. O., Sleeman, K. E., Higginson, I. J., Walshe, C., & CovPall Study, T. (2022). Experiences of staff providing specialist palliative care during COVID-19: A multiple qualitative case study. *Journal of the Royal Society of Medicine*, *115*(6), 220-230. https://doi.org/10.1177/01410768221077366

Carnevale, F. A. (2020). Moral distress in the ICU: It's time to do something about it! *Minerva Anestesiologica*, *86*(4), 455-460. https://doi.org/10.23736/s0375-9393.19.14021-7

Dorman, J. D., & Bouchal, S. R. (2020). Moral distress and moral uncertainty in medical assistance in dying: A simultaneous evolutionary concept analysis. *Nursing Forum*, *55*(3), 320-330. https://doi.org/10.1111/nuf.12431

Dressler, G., Garrett, S. B., Hunt, L. J., Thompson, N., Mahoney, K., Sudore, R. L., Ritchie, C. S., & Harrison, K. L. (2021). "It's case by case, and it's a struggle": A qualitative study of hospice practices, perspectives, and ethical dilemmas when caring for hospice enrollees with full-code status or intensive treatment preferences. *Journal of Palliative Medicine*, *24*(4), 496-504. https://doi.org/10.1089/jpm.2020.0215

Elmore, J., Wright, D. K., & Paradis, M. (2018). Nurses' moral experiences of assisted death: A meta-synthesis of qualitative research. *Nursing Ethics*, *25*(8), 955-972. https://doi.org/10.1177/0969733016679468

Falco-Pegueroles, A., Bosch-Alcaraz, A., Terzoni, S., Fanari, F., Viola, E., Via-Clavero, G., Hoyo, S. G., Parini, A. M., Poveda-Moral, S., Parozzi, M., Guardia-Olmos, J., & Bonetti, L. (2023). COVID-19 pandemic experiences, ethical conflict and decision-making process in critical care professionals (Quali-Ethics-COVID-19 research part 1): An international qualitative study. *Journal of Clinical Nursing*, *32*(15-16), 5185-5200. https://dx.doi.org/10.1111/jocn.16633

Fantus, S., Greenberg, R. A., Muskat, B., & Katz, D. (2017). Exploring moral distress for hospital social workers. *British Journal of Social Work*, *47*(8), 2273-2290. https://doi.org/10.1093/bjsw/bcw113

Fortier, E. P. (2018). *Moral agency and moral distress among registered nurses: From novice to expert* [The University of Regina (Canada)]. 146.

Funk, L. M., Peters, S., & Roger, K. S. (2017). The emotional labor of personal grief in palliative care: Balancing caring and professional identities. *Qualitative Health Research*, *27*(14), 2211-2221. https://doi.org/10.1177/1049732317729139

Gebreheat, G., & Teame, H. (2021). Ethical challenges of nurses in COVID-19 pandemic: Integrative review. *Journal of Multidisciplinary Healthcare, 14*, 1029-1035. https://doi.org//10.2147/jmdh.S308758

Glasdam, S., Ekstrand, F., Rosberg, M., & van der Schaaf, A. M. (2020). A gap between the philosophy and the practice of palliative healthcare: Sociological perspectives on the practice of nurses in specialised palliative homecare. *Medicine Health Care and Philosophy*, *23*(1), 141-152. https://doi.org/10.1007/s11019-019-09918-2

Hold, J. L. (2017). A good death: Narratives of experiential nursing ethics. *Nursing Ethics*, *24*(1), 9-19. https://doi.org/10.1177/0969733015602051

Jerpseth, H., Dahl, V., Nortvedt, P., & Halvorsen, K. (2017). Nurses' role and care practices in decision-making regarding artificial ventilation in late stage pulmonary disease. *Nursing Ethics*, *24*(7), 821-832. https://doi.org/10.1177/0969733015626600

Ko, H. K., Chin, C. C., & Hsu, M. T. (2018). Moral distress model reconstructed using grounded theory. *Journal of Nursing Research*, *26*(1), 18-26. https://doi.org/10.1097/JNR.0000000000000189

Kopchek, L. (2020). *How do palliative care registered nurses apply concepts of ethical decision-making when caring for patients who request medical assistance in dying? An interpretive descriptive qualitative study* [University of Windsor (Canada)]. ProQuest Dissertations & Theses Global.

Laugerat, C., Bouti, C., Lecomte, S., Pilon, N., Turzan, O., Mallet, D., Fradin, S., & Chaumier, F. (2023). La place de l'infirmière d'équipe mobile de soins palliatifs dans la crise COVID-19 [The role of the palliative care mobile team nurse during the COVID-19 crisis]. *Médecine Palliative*, *22*(1), 34-41. https://doi.org/10.1016/j.medpal.2022.08.006

Lim, A., & Kim, S. (2021). Nurses' ethical decision-making during end of life care in South Korea: A cross-sectional descriptive survey. *BMC Medical Ethics*, *22*(1), 9, Article 94. https://doi.org/10.1186/s12910-021-00665-9

Lokker, M. E., Swart, S. J., Rietjens, J. A. C., van Zuylen, L., Perez, R., & van der Heide, A. (2018). Palliative sedation and moral distress: A qualitative study of nurses. *Applied Nursing Research*, *40*, 157-161. https://doi.org/10.1016/j.apnr.2018.02.002

Ma, K., Wright, D. K., Vanderspank-Wright, B., Peterson, W. E., & Carnevale, F. A. (2020). Nurses' moral experiences of ethically meaningful end-of-life care: Distress, resilience, responsibility, and care. *Research and Theory for Nursing Practice*, *34*(3), 269-285. https://doi.org/10.1891/rtnp-d-19-00114

McMillan, K., Wright, D. K., McPherson, C. J., Ma, K., & Bitzas, V. (2021). Visitor restrictions, palliative care, and epistemic agency: A qualitative study of nurses' relational practice during the coronavirus pandemic. *Global Qualitative Nursing Research*, *8*, 12. https://doi.org/10.1177/23333936211051702

Meeker, M. A., & White, D. (2021). Transition to comfort-focused care: Moral agency of acute care nurses. *Nursing Ethics*, *28*(4), 529-542. https://doi.org/10.1177/0969733020952128

Mowat, R., Cook, C., Chapman, M. K., & Roskruge, M. (2023). Good death disrupted: Nurses' moral emotions navigating clinical and public health ethics during the first wave of COVID-19 pandemic. *Journal of Clinical Nursing*, *32*(17-18), 6611-6621. https://doi.org/10.1111/jocn.16702

O'Mathúna, D., Smith, J., Zadvinskis, I. M., Monturo, C., Kelley, M. M., Tucker, S., Miller, P. S., Norful, A. A., Zellefrow, C., & Chipps, E. (2023). Ethics and frontline nursing during COVID-19: A qualitative analysis. *Nursing Ethics*, *30*(6), 803-821. https://doi.org/10.1177/09697330221143150

Panozzo, S., Bryan, T., Collins, A., Marco, D., Lethborg, C., & Philip, J. A. (2020). Complexities and constraints in end-of-life care for hospitalized prisoner patients. *Journal of Pain and Symptom Management*, *60*(5), 984-991. https://doi.org/10.1016/j.jpainsymman.2020.05.024

Peter, E., Simmonds, A., & Liaschenko, J. (2018). Nurses' narratives of moral identity: Making a difference and reciprocal holding. *Nursing Ethics*, *25*(3), 324-334. https://doi.org/10.1177/0969733016648206

Prompahakul, C., & Epstein, E. G. (2020). Moral distress experienced by non-Western nurses: An integrative review. *Nursing Ethics*, *27*(3), 778-795. https://doi.org/10.1177/0969733019880241

Rainer, J., Schneider, J. K., & Lorenz, R. A. (2018). Ethical dilemmas in nursing: An integrative review. *Journal of Clinical Nursing*, *27*(19-20), 3446-3461. https://doi.org/10.1111/jocn.14542

Ramvi, E., & Ueland, V. I. (2019). Between the patient and the next of kin in end-of-life care: A critical study based on feminist theory. *Nursing Ethics*, *26*(1), 201-211. https://doi.org/10.1177/0969733016688939

Reed, F. M., Fitzgerald, L., & Bish, M. R. (2018). A practice model for rural district nursing success in end‐of‐life advocacy care. *Scandinavian Journal of Caring Sciences*, *32*(2), 746-755. https://doi.org/10.1111/scs.12505

Rodney, P. A. (2017). What we know about moral distress. *American Journal of Nursing*, *117*(2), S7-S10. https://doi.org/10.1097/01.Naj.0000512204.85973.04

van den Bosch, G., van Schaik, M., Pasman, H. R., Janssens, R., Widdershoven, G., & Metselaar, S. (2023). Moral challenges of nurses and volunteers in Dutch palliative care. A qualitative study. *Journal of Palliative Care*, *38*(3), 364-371. https://doi.org/10.1177/08258597221098129

Variath, C., Peter, E., Cranley, L., & Godkin, D. (2022). Health care providers' ethical perspectives on waiver of final consent for Medical Assistance in Dying (MAiD): A qualitative study. *BMC Medical Ethics*, *23*(1), Article 8. https://doi.org/10.1186/s12910-022-00745-4

Voultsos, P., Zymvragou, C.-E., & Raikos, N. (2023). Perceptions and experiences of female nurses when confronted with expressing a conscientious objection towards end-of-life care in Greece. *BMC Nursing*, *22*(1), Article 372. https://doi.org/10.1186/s12912-023-01555-8

Wright, D. K., Chan, L. S., Fishman, J. R., & Macdonald, M. E. (2021). "Reflection and soul searching": Negotiating nursing identity at the fault lines of palliative care and medical assistance in dying. *Social Science & Medicine*, *289*, Article 114366. https://doi.org/10.1016/j.socscimed.2021.114366

Wright, D. K., Gastmans, C., Vandyk, A., & de Casterle, B. D. (2020). Moral identity and palliative sedation: A systematic review of normative nursing literature. *Nursing Ethics*, *27*(3), 868-886. https://doi.org/10.1177/0969733019876312
